# Epidemiology of rare diseases in Brazil: protocol of the Brazilian Rare Diseases Network (RARAS-BRDN)

**DOI:** 10.1186/s13023-022-02254-4

**Published:** 2022-02-24

**Authors:** Têmis Maria Félix, Bibiana Mello de Oliveira, Milena Artifon, Isabelle Carvalho, Filipe Andrade Bernardi, Ida V. D. Schwartz, Jonas A. Saute, Victor E. F. Ferraz, Angelina X. Acosta, Ney Boa Sorte, Domingos Alves, RARAS Network group, Tatiana Amorim, Gisele Maria Araujo Felix Adjuto, Rosemarie Elizabeth Schimidt Almeida, Flávia Resedá Brandão, Larissa Souza Mario Bueno, Maria Denise Fernandes Carvalho de Andrade, Cristina Iacovelo Cagliari, Maria Terezinha Cardoso, Ellaine Doris Fernandes Carvalho, Marcela Câmara Machado Costa, Antonette El-Husny, Lavinia Schuler Faccini, Rodrigo Ambrosio Fock, Rodrigo Neves Florêncio, Marcial Francis Galera, Roberto Giugliani, Liane de Rosso Giuliani, Anette S. Grumach, Dafne G. Horovitz, Juan Clinton Llerena-Junior, Chong A. E. Kim, Rayana Elias Maia, Ana Maria Martins, Paula Frassinetti Vasconcelos de Medeiros, Nina Rosa de Castro Musolino, Marcelo Eidi Nita, Henrique Gil da Silva Nunesmaia, Jose Carlison Santos de Oliveira, Wagner José Martins Paiva, Helena Pimentel, Louise Lapagesse de Camargo Pinto, Vânia Mesquita Gadelha Prazeres, Betânia de Freitas Rodrigues Ribeiro, Erlane Ribeiro, Márcia Maria Jardim Rodrigues, Maria José Sparça Salles, Maria Teresa Vieira Sanseverino, Eliane Pereira dos Santos, Mara Lucia Schmitz Ferreira Santos, Flávia Mori Sarti, Luiz Carlos Santana da Silva, Raquel Tavares Boy da Silva, Carlos Eduardo Steiner, Ana Beatriz Winter Tavares, Thais Bonfim Teixeira, Alberto Vergara, Paulo Ricardo Gazzola Zen, Marcos Guimarães Zuchetti

**Affiliations:** 1 Medical Genetics Service, Porto Alegre Clinical Hospital, Ramiro Barcelos St., 2350, Porto Alegre, RS 90035-903 Brazil; 2 Federal University of Rio Grande do Sul, Porto Alegre, Brazil; 3 Department of Social Medicine, Ribeirão Preto Faculty of Medicine, University of São Paulo, São Paulo, Brazil; 4 Department of Genetics, Ribeirão Preto Faculty of Medicine, University of São Paulo, São Paulo, Brazil; 5 Department of Pediatrics, Federal University of Bahia, Salvador, Brazil

**Keywords:** Rare diseases, Epidemiology, Healthcare system, Public health

## Abstract

The Brazilian Policy of Comprehensive Care for People with Rare Diseases (BPCCPRD) was established by the Ministry of Health to reduce morbidity and mortality and improve the quality of life of people with rare diseases (RD). Several laboratory tests, most using molecular genetic technologies, have been incorporated by the Brazilian Public Health System, and 18 specialised centres have so far been established at university hospitals (UH) in the capitals of the Southern, Southeastern and Northeastern regions. However, whether the available human and technological resources in these services are appropriate and sufficient to achieve the goals of care established by the BPCCPRD is unknown. Despite great advances in diagnosis, especially due to new technologies and the recent structuring of clinical assessment of RD in Brazil, epidemiological data are lacking and when available, restricted to specific disorders. This position paper summarises the performance of a nationally representative survey on epidemiology, clinical status, and diagnostic and therapeutic resources employed for individuals with genetic and non-genetic RD in Brazil. The Brazilian Rare Disease Network (BRDN) is under development, comprising 40 institutions, including 18 UH, 17 Rare Diseases Reference Services and five Newborn Screening Reference Services. A retrospective study will be initially conducted, followed by a prospective study. The data collection instrument will use a standard protocol with sociodemographic data and clinical and diagnostic aspects according to international ontology. This great collaborative network is the first initiative of a large epidemiological data collection of RD in Latin America, and the results will increase the knowledge of RD in Brazil and help health managers to improve national public policy on RD in Brazil.

## Background

The term rare disease (RD) is used to describe disorders that affect a small percentage of the population when compared to prevalent disorders in the general population. They are considered chronic and disabling, affecting the quality of life of individuals and their families. No universal definition exists for RD [1]. Despite being individually rare, they collectively affect 10% of the population, significantly affecting the health system. The diagnosis, management and treatment of RD must be recognised by healthcare professionals [2]. An estimated 5000–8000 RD have been described, 80% with a genetic aetiology [3, 4].

## Rare diseases in Brazil

Brazil is the fifth-largest country in the world, with an area of 8,516,000 km^2^ and an estimated population of 211.8 million in July 2020. The Brazilian Ministry of Health defines RD according to the World Health Organization (WHO) as those affecting 65 to 100,000 individuals [5]. In Brazil, RD has been diagnosed and treated at specialised centres of medical genetics services at university hospitals (UH) and Newborn Screening Reference Services (NSRS) for specific disorders diagnosed by neonatal screening programs. These centres, located primarily in large cities and state capitals, have offered RD diagnosis using clinical and mainly research laboratories because the majority of genetic tests have not been offered by the Brazilian Unified Health System (Sistema Único de Saúde; SUS). In January 2014, the Ministry of Health established the Brazilian Policy of Comprehensive Care for People with Rare Diseases (BPCCPRD) [5]. This policy aims to reduce morbimortality and secondary manifestations and improve the quality of life of people with RD by promoting prevention and earlier detection, allowing opportunities for treatment, decreasing incapacity and promoting palliative care. The organisation of care for people with RD in Brazil is structured in two main axes: RD of genetic origin, including congenital anomalies and late-onset disorders, intellectual disabilities and inborn errors of metabolism [5]; and RD of non-genetic origin, including infectious diseases, inflammatory and autoimmune disorders [2]. Therefore, the Rare Disease Specialized Care Services and Rare Disease Reference Services (RDRS) were established, and a list of genetic tests was incorporated into the SUS.

Since the publication of the Policy, 18 RDRS have been implemented at UH and other facilities. However, a delay from implementation to actual assistance of patients was observed in the majority of services, with only 10 institutions reporting consultations and procedures to the Ministry of Health as of July 2020. Considering the Brazilian territory and population, insufficient facilities exist to attend to the RD population. Consequently, an enormous barrier to access to care still leads to delayed diagnosis, limited access to resources and treatment, and delayed management [3, 6].

Specific treatments now exist for some RD such as inborn errors of metabolism (enzyme replacement therapy, diet therapy and supplementation of enzymatic cofactors), increasing the life expectancy and quality of life and decreasing mortality of some individuals. Recently, the use of advanced therapies has become a reality in several disorders [7]. For this, early diagnosis is fundamental in decreasing the saga experienced by people with RD and their families. This impacts the health system due to the high costs of therapies and diagnostic methods [8].

In this context, the lack of accurate epidemiological data on RD hinders the estimation of the budgetary impact of these new technologies to drive the decision-making of health managers. In the research priority agenda of the Brazilian Ministry of Health, post-incorporation evaluation is necessary to monitor the real-world effectiveness of each novel advanced therapy in the Brazilian health system context [9]. Therefore, the SUS is limited in this continental country. Considering the synergy of pharmacological therapy, access to early diagnosis, rehabilitation and general care could provide results to match the more controlled environment of randomised studies.

However, the great majority of RD will not have any specific treatment in the next few years, and those disorders also must be prioritised. A series of measures apply to every RD of genetic origin, with potential prevention through genetic counselling, therapy and multidisciplinary treatment (such as physical therapy, speech pathology and dietotherapy) increasing the quality of life and life expectancy.

Furthermore, compared to other diseases, less information on the clinical and economic burden of RD exists for use in the care management process, diagnosis and therapeutic management, as well as the administrative and financial management of the institutions that attend those patients.

RD are highly diverse, but similar issues could be used and explored with a collaborative approach, sharing knowledge and experience to develop a common system model of several disorders [10].

## Epidemiology of rare disease in Brazil

Despite great advances in diagnosis, especially due to new technologies and the recently approved policy for the care of RD in Brazil, epidemiological data are lacking. Without national epidemiological data, the data available in the literature are restricted to specific disorders or regions due to the efforts of the scientific community [11–28] as shown in Table 1.

**Table 1:** National and international studies containing original epidemiological data on the incidence and/or prevalence of RD in Brazil

References	Title	Clinical condition	Region	Rate
Costa-Motta et al. [10]	A community-based study of mucopolysaccharidosis type VI in Brazil: the influence of founder effect, endogamy and consanguinity	Mucopolysaccharidosis type VI	Monte Santo, Bahia	Carriers: 41:100
Munford et al. [11]	A genetic cluster of patients with variant xeroderma pigmentosum with two different founder mutations	Variant xeroderma pigmentosum	Goias	17:1000
Leadley et al. [12]	A systematic review of the prevalence of Morquio A syndrome: challenges for study reporting in rare diseases	Morquio A syndrome	International	1:1,179,000
Souza et al. [13]	BH4 deficiency identified in a neonatal screening program for hyperphenylalaninemia	BH4 deficiency	Minas Gerais	2.1:1,000,000
Cardoso et al. [14]	Clusters of genetic diseases in Brazil	Rare diseases	Brazil	1:235,000
Colombo et al. [15]	Epidemiology of candidemia in Brazil: a nationwide sentinel surveillance of candidemia in eleven medical centers	Candidemia	Brazil	2.49:1,000 admissions; 0.37:1,000 patient-days*
Khan et al. [16]	Epidemiology of mucopolysaccharidoses	Mucopolysaccharidoses	International	1.04:100,000
de Azevedo Medeiros et al. [17]	High prevalence of Berardinelli-Seip Congenital Lipodystrophy in Rio Grande do Norte State, Northeast Brazil	Berardinelli-Seip	Rio Grande do Norte	3.23:100,000
Walker et al. [18]	Huntington's disease-like disorders in Latin America and the Caribbean	Huntington's disease-like disorders	Rio Grande do Sul	1.85:100,000
Hamerschlak et al. [19]	Incidence and risk factors for agranulocytosis in Latin American countries–the Latin Study: a multicenter study	Agranulocytosis	Latin America	0.35:1,000,000 inhabitant–years*
Hamerschlak et al. [20]	Incidence of aplastic anemia and agranulocytosis in Latin America—the LATIN study	Aplastic anemia and agranulocytosis	International	Aplastic anemia: 2.7:1,000,000 per year* Agranulocytosis: 0.5:1,000,000 per year*
Raskin et al. [21]	Incidence of cystic fibrosis in five different states of Brazil as determined by screening of p.F508del, mutation at the CFTR gene in newborns and patients	Cystic fibrosis	Brazil	1:7576*
Bustamante-Teixeira et al. [22]	Incidence of rare cancers in the city of São Paulo, Brazil	Rare cancers	Sao Paulo	365:100,000*
Wagner et al. [23]	Neonatal screening for hemoglobinopathies: results of a public health system in South Brazil	Sickle cell disorder	Rio Grande do Sul	1:9120*
Botler et al. [24]	Phenylketonuria, congenital hypothyroidism and haemoglobinopathies: public health issues for a Brazilian newborn screening program	Phenylketonuria, congenital hypothyroidism and haemoglobinopathies	Rio de Janeiro	Sickle cell disease: 1:1,28* Congenital hypothyroidism: 1:1,030* Phenylketonuria: 1:28,427 to 1:16,522*
Boton Pereira et al. [25]	Primary Immunodeficiencies in a Mesoregion of São Paulo, Brazil: Epidemiologic, Clinical, and Geospatial Approach	Primary Immunodeficiencies	Presidente Prudente mesoregion	0.59:10,000*
Balmant et al. [26]	Rare cancers in childhood and adolescence in Brazil: First report of data from 19 population-based cancer registries	Rare cancers	Brazil	Birth to 9 years: 5.19:1,000,000 10–14 years: 15.60:1,000,000 15–19 years: 29.72:1,000,000
Orioli et al. [27]	Sirenomelia: an epidemiologic study in a large dataset from the International Clearinghouse of Birth Defects Surveillance and Research, and literature review	Sirenomelia	International	0.98:100,000

*Incidence

A rare exception is the National Neonatal Screening Program (Programa Nacional de Triagem Neonatal), with high coverage of all regions of Brazil [29]. This public health program screens newborns for phenylketonuria, congenital hypothyroidism, cystic fibrosis, congenital adrenal hyperplasia, biotinidase deficiency and sickle cell disease. Despite this, epidemiological data on these diseases are still scarce [30], and a national database is not available for broad access.

After implementation of the BPCCPRD, epidemiological data can be drawn based on more than 14,700 consultation and diagnostic procedures performed from 2017 to June 2020 in 10 of the 17 RDRS [31]. In this database, data are registered for all procedures using the International Statistical Classification of Diseases and Related Health Problems, 10^th^ Revision (ICD-10). This is not the optimal source of identification for RD because many different disorders are grouped in single codes. Data available for the most common diagnoses in the last 4 years of the BPCCPRD are shown in Table 2. However, the data refer to procedures performed and not the number of patients attended at the institutions because an individual may have up to three procedures. Importantly, these data do not include cases diagnosed by neonatal screening programs because the majority of RDRS are not Neonatal Screening Reference Services (NSRS).

**Table 2:** Number of procedures performed by RDRS since implementation and pathologies most frequently attended by the centers (2016–2020)

Demographic region	South			Southeast		Northeast		Midwest	
Institution	HPP	HCPA	FMABC	Unicamp	IFF	APAE-Salvador	HIAS	APAE- Anápolis	HAB
Date of implementation	Oct, 2016	Dec, 2016	Dec, 2019	Nov, 2016	Dec, 2016	Jul., 2018	Dec, 2019	Oct, 2016	Dec, 2016
Number of Procedures	5131	11,132	1264	384	863	111	51	361	3140
Most frequent disorders registered (ICD10–name–number–%)	ID (F71.0, F70.0, F72.0, F79.0–1180–22.99)	SCA (G11.2–924–8.3)	Unspecified ID(F79.0–293–23.18)	Unspecified ID (F79.0, F79.9–55–14.32)	OI (Q78.0–224–25.95)	Moderate ID(F71.0–80–72.072)	Craniofacial malformation syndromes (Q87.0–9–17.64)	Other malformations (Q87.8–135–37.39)	ID (F79.0, F70.0, F71.0–745–23.72)
	Syndromic malformations (352–6.86)	NF1 (Q85.0–916–8.22)	Wiskott-Aldrich syndrome (D82.0–190- 15.03)	Di George Syndrome (D82.1–31–8.07)	Syndromic malformations (Q87.8–105–12.16)	Severe ID (F72.0–12–10.81)	Neurodegenerative diseases (G31.8–6–11.76)	ID (F79.0–68–18.83)	Motor neuron diseases (G12.2–254–8.08)
	Mitochondrial myopathy (343–6.68)	OI (Q78.0–718–7.34)	Moderate ID(F71.0–68–5.37)	Chromosomal anomalies (Q98.9–29–7.55)	Other ID (F78.0–91–10.54)	Chromosomal abnormality, unspecified (Q99.9–8–7.20)	Other malformations (Q87.8–6–11.76)	Chromosomal anomalies (Q99.9–38–10.52)	Classic Phenylketonuria (E70.0–195–6.21)
	Chromosomal anomalies (Q99.9–192–3.74)	Muscular dystrophy (G71.0–650–5.83)	Mild ID (F70.0–65–5.14)	Defects in the complement system (D84.1–23–5.98)	Other skeletal malformations (Q87.5–74–8.57)		Muscular dystrophy (G71.0–6–11.76)	Rett syndrome (F84.2–10–2.77)	Disorders of plasma-protein metabolism (E88.0–176–5.60)
	Quimera 46 XX/46 XY (Q99.0–131–2.55)	Hereditary spastic paraplegia (G11.4–523–4.69)	Muscular dystrophy (G71.0–54–4.27)	Craniofacial malformation syndromes (Q87.0–19–4.94)				Other demyelinating diseases of CNS (G37.8–10–2.77)	Disorders of aminoacids metabolism (E72.9–162–5.15)
	Other chromosomal anomalies (Q99.8–112–2.18)	Other sphingolipidosis (E75.2–460–4.13)		Turner syndrome (Q96.0–16–4.16)					Disorders of galactose metabolism (E74.2–135–4.29)
	Enlers-Danlos syndrome (Q79.6–90–1.75)	Craniofacial malformation syndromes(Q87.0–380 -3.41)		Congenital malformation syndromes predominantly associated with short stature (Q87.1–14–3.64)					Other disorders of mineral metabolism (E83.8–131–4.17)
	Glycogen storage disease (E74.0–77–1.50)	Neurodegenerative diseases (g31.8–356–3.19)		Other malformations (Q87.8–12–3.125)					
	Other phakomatoses (Q85.8–69–1.34)	Unspecified ID (F79.0–351–3.15)							
	Disorders of amino acid transport (E72.0–68–1.32)	Hereditary motor and sensory neuropathy (G60.0–333–2.99)							

HCPA: Hospital de Clínicas de Porto Alegre; HPP: Hospital Pequeno Príncipe; FMABC: Faculdade de Medicina do ABC; IFF: Instituto Nacional de Saúde da Mulher, da Criança e do Adolescente Fernandes Figueira; APAE: Associação de Pais e Amigos dos Excepcionais; HIAS: Hospital Infantil Albert Sabin; HAB: Hospital de Apoio de Brasília; OI: Osteogenesis imperfecta; SCA: Spinocerebellar ataxias; NF1: Neurofibromatosis type 1; ID: Intellectual disability

## The importance of an epidemiologic study

One of the great challenges associated with RD worldwide is the inadequacy of medical systems to diagnose these disorders correctly and promptly, leading to a delay in management or therapy. A study in the USA showed a mean time of 7 years for diagnosis of RD, causing anxiety, financial difficulties for families and increasing morbidity [32]. In developing countries, this delay is even longer [33]. For example, a Brazilian study on mucopolysaccharidoses showed a delay of 4.8 years between the onset of signs and symptoms and diagnosis [34]. Another problem faced by people with RD is that even after receiving a specific and correct diagnosis, they might have limited access to resources at specialised centres, coordinated assistance, patient support and appropriate treatment. For several RD, no specific treatment exists, and information on progression or prognosis is limited. Therefore, research on the natural history and pathophysiological mechanisms of RD is necessary to develop specific therapies [3].

Population-based research in RD is difficult due to the low prevalence of these disorders and the high costs of studies [35]. Therefore, the availability of reliable epidemiological data on RD is a crucial and urgent unmet need. The European Union Council recognised in 2009 the importance of supporting networks, registries and databases on specific RD. These registries are a powerful tool to help to develop clinical research, clinical trial planning, better assistance to patients and support to health management [36].

Estimating the global prevalence of RD is a great challenge due to the diversity of data collected by a variety of sources, including published case reports and systematic reviews, patient registries and specialist boards. This is aggravated by the use of several different study methods and the lack of diagnostic criteria or codification systems used to capture the data. The nature of RD, having small numbers of cases and clinical heterogeneity, can compromise the data. Further, several disorders vary by geographical area due to the diversity of the population and environmental and social pressures. The necessity of health indicators for RD has been recognised to evaluate health status and results and to monitor the efficacy of health initiatives and policies. The use of data registries dedicated to one or more RD has been identified as strategically important to guarantee the availability of health indicators. Additionally, a national database for RD should be integrated with other existing databases [37].

In practice, this can be done for specific disorders where a good-quality database is already in place. Therefore, a broader approach is necessary to produce health indicators that correspond to the majority of RD. For this goal, a population database is important [38].

In this context, knowing the magnitude of RD in Brazil, a national survey on these conditions can provide important information about their profile to expand knowledge about epidemiology, clinical and diagnostic aspects and therapeutic itineraries [39].

This position statement aims to report the Brazilian Rare Disease Network (BRDN) initiative. This is a project funded by the Ministry of Health of Brazil through the National Council for Scientific and Technological Development (Conselho Nacional de Desenvolvimento Científico e Tecnológico; CNPq). The main objective of this study is to perform an inquiry into epidemiology, clinical findings, diagnostic and therapeutic resources, and costs of RD in Brazil.

## Description

**Fig. 1:**
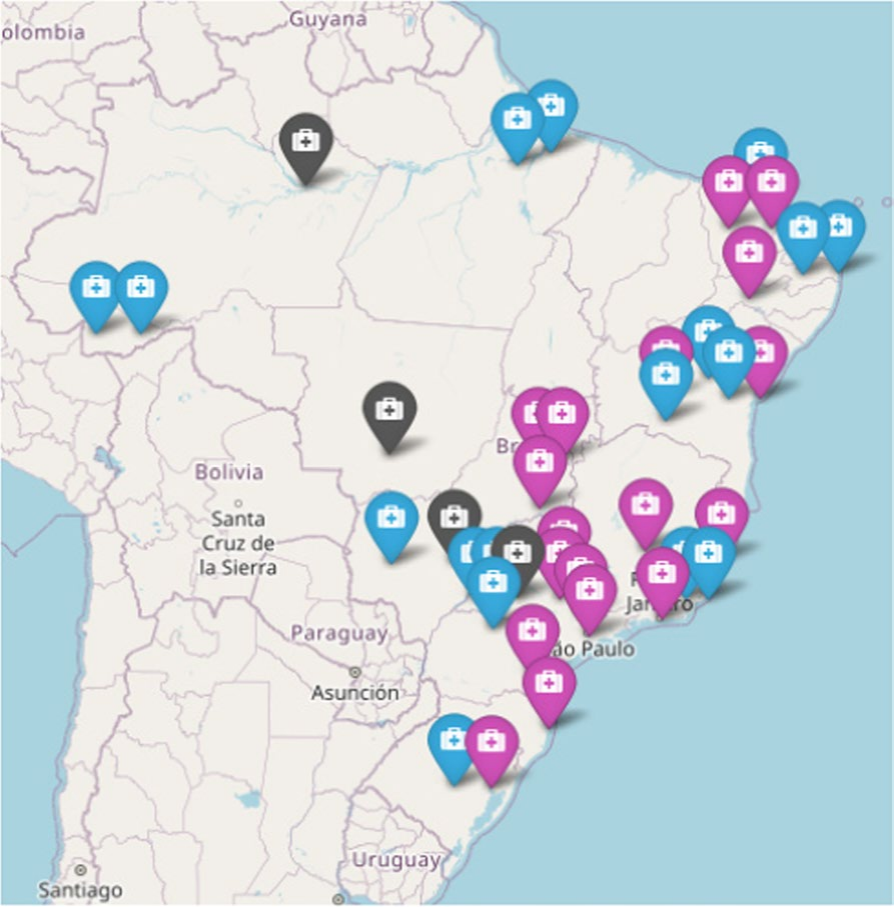
Brazilian Rare Disease Network: Brazilian map points the location and distribution of participant institutions (pink: RDRS; blue: UH; black: NSRS)

1. Ethical considerations
  The BRDN project was approved in notice nº. 25/2019 by CNPq with financial support from the Ministry of Health of Brazil. This project was submitted and approved by the Institutional Ethics Committee Board of Porto Alegre Clinical Hospital, the coordinator centre (CAAE: 33970820.0.1001.5327) and has been submitted and approved by all participant institutions IRB.
2. Brazilian Rare Disease Network
  A population census study (survey) will be developed to collect ambispective (retrospective and prospective) data, coupled with an innovation proposal for the creation of a service network involving several institutions throughout the national territory. The first step is the consolidation of the BRDN, which is already underway. The articulation and construction of the structured network includes 40 voluntary institutions that provide RD diagnosis and treatment in Brazil: all 17 RDRS, five NSRS and 18 UH. All institutions are spread across the country and in all Brazilian regions, as seen in Fig. 1. The network was established through contact with experts from the Brazilian Medical Genetics Society and Brazilian Neonatal Screening and Inborn Errors of Metabolism Society and several additional institutions that assist specific RD. Data management will be performed by the Ribeirão Preto Faculty of Medicine. The protocol steps are summarised in Fig. 2.

**Fig. 2:**
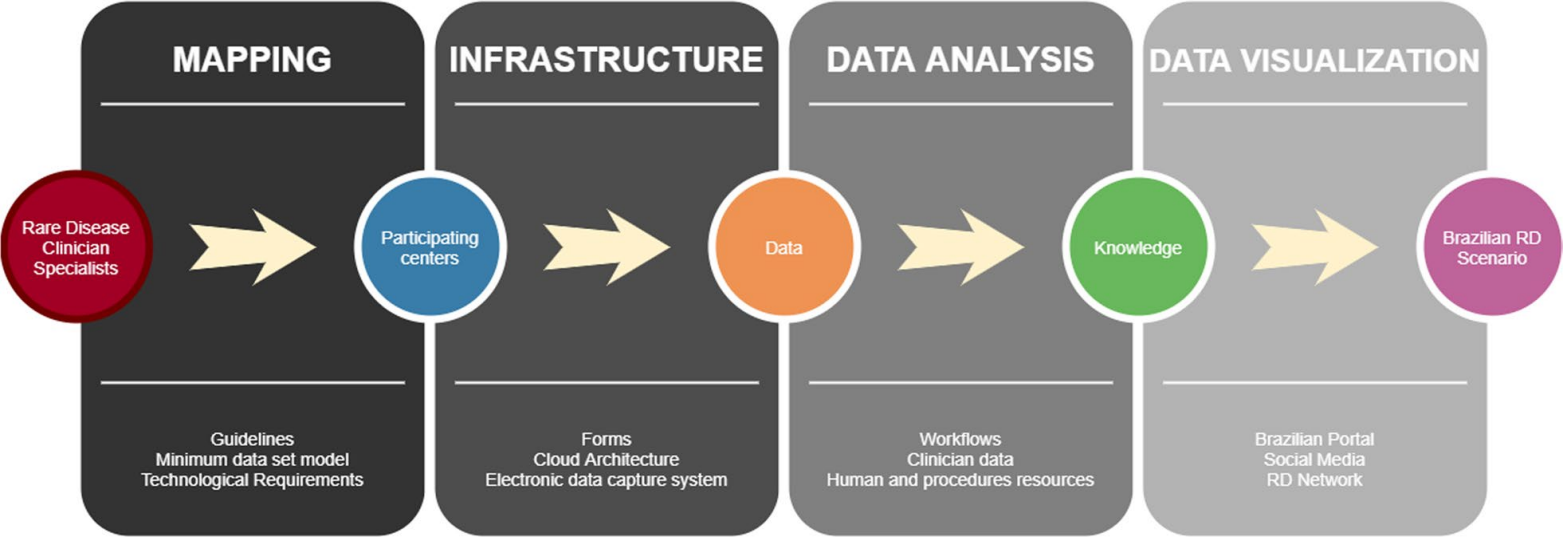
Summary of data collection and analysis
3. Laboratory and human resources
  A survey using an electronic form has been designed to collect data on the technology laboratory resources and human resources available at the participant institutions for diagnosis and management of RD. Data governance was described elsewhere [40]. Since the creation of the BPCCPRD in 2014, the SUS has incorporated 19 diagnostic tests, in addition to organising the assistance network [41]. Data on the type and number of laboratory analyses used for RD diagnosis will be collected as karyotype, fluorescence in situ hybridization, chromosomal microarray, Southern blot, MLPA, PCR or qPCR, Sanger sequencing, next-generation sequencing panels, exome sequencing, carnitine and carnitine profile, amino acid, organic acid and enzymatic analysis. The number of different professionals for diagnosis and management of RD at each institution will also be collected.
4. Retrospective epidemiological data
  Retrospective data will be collected. Paper or electronic medical charts of all cases assisted at the institutions from 2018 to 2019 will be reviewed.
5. Data collection
  Data collection will follow a standard protocol designed specifically for this study by a group of experts. This instrument will collect date of birth, race, date of the first appointment at the institution, age, consanguinity, birthplace, city, diagnosis, aetiological diagnosis methods (clinical, biochemical or cytogenetic/ molecular), source of reimbursement for laboratory exams (SUS, insurance, out of pocket, research or pharmaceutical companies), time of diagnosis (prenatal, postnatal or neonatal screening test), age at first symptoms, phenotype (described using at least five Human Phenotype Ontology terms for both diagnosed and undiagnosed RD), treatment (specific, dietitian or rehabilitation), previous hospitalisation and vital status (Fig. 3). Information about the diagnosis, if clinically suspected or confirmed by laboratory analysis will be collected. Coding of the disease will also be presented considering the name of the disease, Orpha number, ICD-10 or OMIM classification, allowing comparison with data from other platforms, such as Orphanet. All data will follow the minimum dataset standards of the Ministry of Health.

**Fig. 3:**
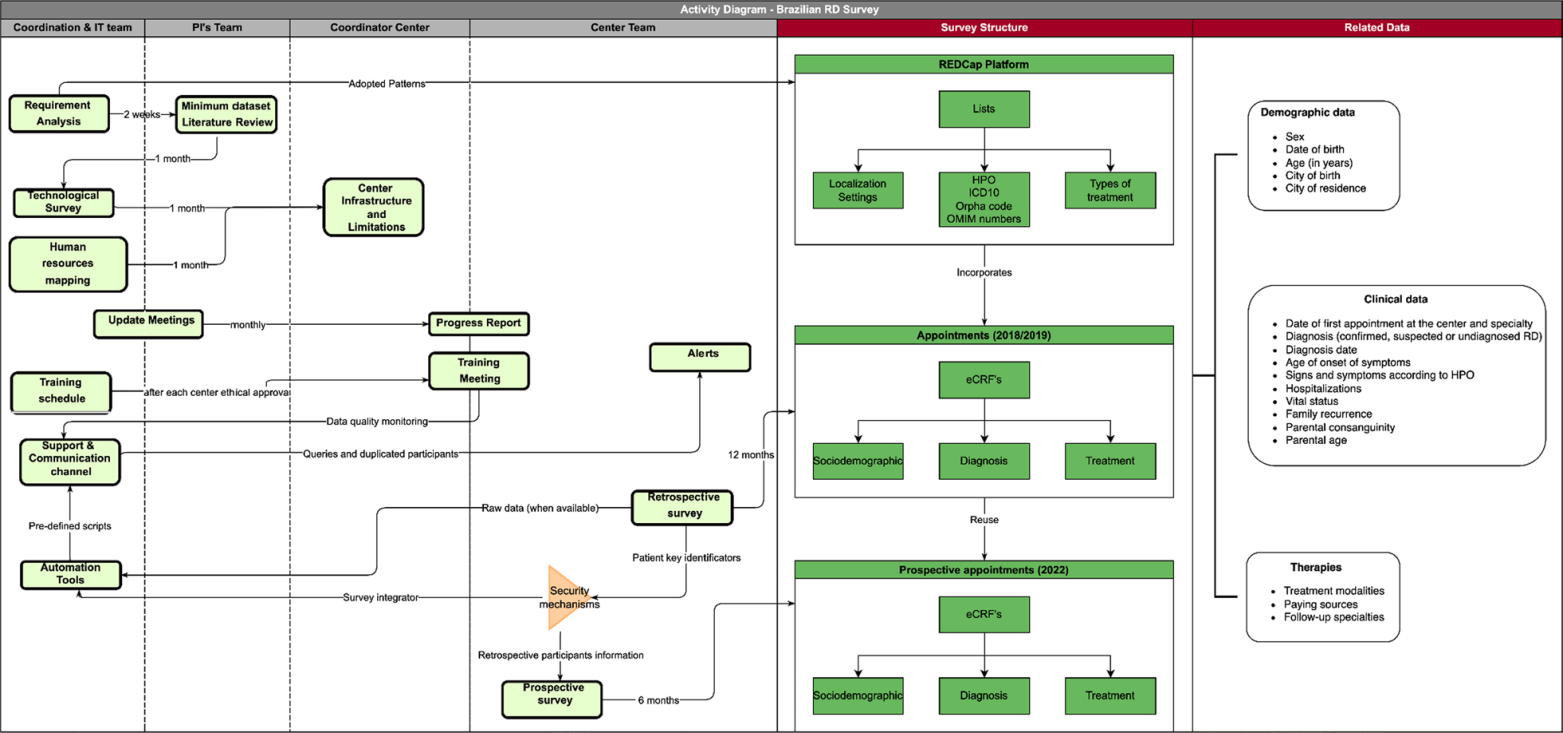
Activity diagram: survey processes, structure and related data
6. An electronic database will be available in a specific server allowing data sharing and networking using a cloud computational tool at InterNuvem from São Paulo University. Infrastructure and analysis data has been published elsewhere [40].
7. A systematic audit of the data collected at each centre will be conducted following the protocol established by the FMRP-USP team [40, 42]. A partial report will be produced characterising the population by region and characterising the centres by their maturity in data collection. We emphasise that articulation between the services related to diagnosis and care for these individuals, through the formation of a standardised and national database and consolidating a network of hospitals and services trained to care for RD, increases the possibility that the result of the survey can be magnified as a real support for the BPCCPRD. In addition, even the difficulties that this study may encounter related to the quality of data and retrospective records will allow a broad view of the healthcare of people with RD in the SUS, highlighting regional particularities and healthcare system asymmetries. This will enable rational planning to optimise a universal, comprehensive and equitable healthcare policy for these individuals.
8. Prospective epidemiological data
  In the second phase, an observational prospective cohort study is planned in the same centres, using the same data collection instrument to analyse the impact of this intervention on improving the data quality to characterise individuals with rare genetic and non-genetic diseases in Brazil.
  In this stage, the report characterising the informational maturity of the centres will allow online training directed at the difficulties of each in relation to data collection in the instrument used in the first phase. Data quality indicators will be observed in this intervention to produce a reliable picture of the maturity of data collection on RD in Brazil, mainly in relation to the difficulties encountered in coding the disease.
9. Brazilian Rare Disease Atlas online
  An online Brazilian Rare Disease Atlas will be developed according to WHO guidelines for developing health observatories [43] and the standards of the Ministry of Health DATASUS [44]. This will be available for health professionals, health managers and the general public.
  The atlas will present the following data:
  The number and distribution of cases of RD in the Brazilian territory (according to state and region) and the following items: the name of the disease, Orpha number, ICD-10 and OMIM classification, gene name and symbol, year at diagnosis and sex.
  The number of cases with and without a conclusive diagnosis.
  The locations of health assistance with a specific diagnosis (name of the disorder, Orpha number, ICD-10 and OMIM classification, and gene name and symbol).
  The number of cases with a specific therapy (such as enzymatic replacement, dietotherapy and gene therapy).
  Identification of clusters of RD of genetic origin in Brazil.
10. In the third phase, an assessment of the journey for patients with RD will be conducted with the creation of management dashboards focused on value-based health management [45]. This approach will allow interviewing a proportion of patients during the study – a transversal component. The objective is to ensure a consistent and faithful portrait of patients seen at a referral centre in Brazil, generating information on clinical outcomes centred on patients, such as quality of life and social preferences that will allow the calculation of utility and quality-adjusted life years. These patients will be interviewed by a properly trained health professional, in a structured interview, after signing an informed consent form. A structured questionnaire will be used to collect information related to the patient's diagnosis and treatment journey.
  This stage of the study will have a longitudinal observational design. The data will be collected and questionnaires applied in three moments: visit 1 (month 0), visit 2 (6 months) and visit 3 (12 months).
  The clinical conditions selected for this part of the study follow the Federal Therapeutic Guidelines (Protocolos Clínicos e Diretrizes Terapêuticas) [46–53] and were chosen according to their financial impact, due to either high-cost medications or higher prevalence than other RD. Unlike the previous two stages that cover all RD, this part of the study will focus on the following pathologies: acromegaly (ICD10: E22.0), amyotrophic lateral sclerosis (ICD10: G12.2), classical homocystinuria (ICD10 E72.1), cystic fibrosis (ICD10 E84), Duchenne muscular dystrophy (ICD10: G71.0), familial amyloid polyneuropathy (TTR-FAP; ICD10: E85.1), Gaucher disease (ICD10: E75.2), hereditary angioedema (HAE) caused by C1-esterase inhibitor deficiency (ICD10: D84.1), mucopolysaccharidosis type II (ICD10: E76.1), osteogenesis imperfecta (ICD10: Q78), phenylketonuria (ICD10: E70.0 and E70.1), Prader–Willi syndrome (ICD-10: Q87.1) and spinal muscular atrophy (ICD10: G12.0).

## Plans and goals

The first step was the consolidation of the network. All participating centres were invited to compose the network, and an initial meeting in July 2020 was conducted virtually, due to the COVID-19 pandemic. The centres and their members were presented, in addition to explanations of the network's main objectives. After this initial meeting, monthly meetings have been held with the presence of all participant centres, and the collection forms were discussed and finalised.

The second step consists of a national survey on RD. A population census study (survey) will be developed to collect ambispective (retrospective and prospective) data. The data collection instruments have been built and are in the validation stage. These instruments should serve as a basis for the steps that involve retrospective data collection from partner institutions, as well as a model for the step involving the prospective analysis.

Data from 2018 to 2019 from DATASUS, the informatics department of the SUS [14], demonstrates that 6,495 diagnostic procedures for RD were performed by the first five RDRS that presented production data. Considering that the more robust RDRS attend approximately 1000 patients per year, and the others 500 patients per year, a sample number of approximately 55,000 individuals is estimated.

## Conclusions

The present position statement describes the aims and methodology for epidemiological data collection on RD in Brazil based on the BRDN initiative. The results of this project will impact health policy for RD in Brazil and can serve as an example to collect RD data in Latin America.

## Data Availability

Not applicable.

## Abbreviations

BPCCPRD: Brazilian Policy of Comprehensive Care for People with Rare Diseases
CNPq: Conselho Nacional de Desenvolvimento Científico e Tecnológico
ICD-10: International Statistical Classification of Diseases and Related Health Problems, 10th Revision
IRB: Institutional Review Board
NSRS: Newborn Screening Reference Service
OMIM: Online Mendelian Inheritance in Man
RARAS: Brazilian Network of Rare Diseases
RD: Rare diseases
RDRS: Rare Disease Reference Service
SUS: Brazilian National Health System, Sistema Único de Saúde
UH: University hospitals
WHO: World Health Organization
